# Citing *Phcog Mag*. articles made easy

**DOI:** 10.4103/0973-1296.80661

**Published:** 2011

**Authors:** K. K. Mueen Ahmed

**Affiliations:** *Managing Editor, Pharmacognosy Magazine, Bangalore, India*

I am pleased to introduce the April–June issue to you with very interesting articles related to medicinal plant research. As you are aware, *Phcog Mag*. is one of the most read and favorite journals in the field of pharmacognosy. Since a new electronic submission system was introduced at the end of 2009 (www.journalonweb.com/pm),[1] *Phcog Mag*. has been attempting to improve the editorial process, further shortening the time taken to decide the fate of the submitted articles and to make the process less disturbing for authors. More important, the article quality is being thoroughly checked for each manuscript by peer review process using multiple reviewers. It is arduous to complete the task sometimes.[2] We are striving hard to finish the whole publishing process in the stipulated period of time. Being indexed in Science Citation Index, Scopus, Pubmed and other important databases, it is the most important task for us to have an impact factor (IF) soon.

## CITATIONS AND IMPACT FACTOR

IF is commonly used as a tool for measuring the quality of journals. IFs help us to guide in choosing by determining which journals are most frequently cited. Citation frequency may therefore better reflect the importance of journals to researchers. The use of IF as an index of journal quality relies on the theory that citation frequency accurately measures a journal’s importance to its end users.[3]

Based on the analysis provided by Medknow,[4] unofficial IF is constantly rising every year as we are moving ahead. Tables 1 and 2 show the details of unofficial IF and citation analysis for the year 2009 and 2010. As you can observe, more and more articles are being cited by other researchers and we have added many features in our website to make the citing process much easier.

**Table 1:** Citation analysis for the year 2009

Journal	Citations* in 2009 to		Published in		Impact factor	Citation in 2009 to article of 2009	Articles published in 2009	Immediacy index
	2008	2007	2008	2007				
*Phcog Mag.*	27	0	76	46	0.221	24	97	0.247

* Citations include non-SCI journals also

**Table 2:** Citation analysis for the year 2010

Journal	Citations* in 2010 to		Published in		Impact factor	Citation in 2010 to article of 2010	Articles published in 2010	Immediacy index
	2009	2008	2009	2008				
*Phcog Mag.*	36	27	97	76	0.364	8	62	0.129

* Citations include non-SCI journals also, Courtesy: Medknow Publishers and Media Pvt. Ltd.

## MULTIPLE CITATION FORMAT ENABLED WEBSITE: *PHCOG MAG*.

**Figure 1:**
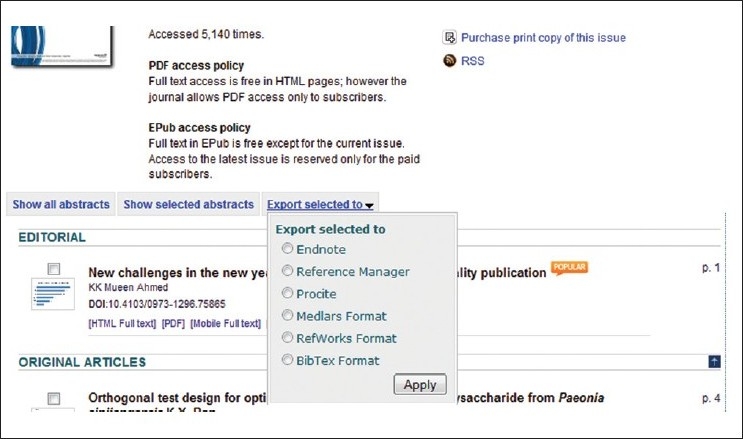
Screenshot from *Phcog Mag*. website for exporting citations of selected articles

Current issue and archives of *Phcog Mag*. are easily available in different citation formats to enable the other researchers to use the most important article published. Multiple citation formats such as Endnote, Reference Manager, Procite, Medlars, Refworks, Bibtex are easily exported from *Phcog Mag*. website (www.phcog.com)[5] with just a simple click [Figure 1].

## CITING *PHCOG MAG*. ARTICLES VIA CROSSREF LINKING

Articles published in *Phcog Mag*. are being assigned with Digital Object Identifier (DOI) with CrossRef to provide reference linking across multiple publishers. CrossRef uses a DOI system to link citations across publishers. Each DOI is associated with a set of basic metadata and a URL pointer to the full text, so that it uniquely identifies the content item and provides a persistent link to its location on the internet. DOIs are alphanumeric strings assigned to digital objects. Each DOI is unique and, once assigned to an item, remains a constant locator, not changing even as an object moves from URL to URL. To find a DOI number of a published article, please use the CrossRef’s free DOI link (look up: http://www.crossref.org/guestquery).[6] Many journals seek DOI in cross-references in order to trace back the original article cited in the manuscript. DOI has been included and displayed in each article published, and it could be easily obtained from our website. Citing linked with DOI enables reliable source of cited source very easily.

## CITING *PHCOG MAG*. ARTICLES AND SYNCHRONIZING WITH ZOTERO

**Figure 2:**
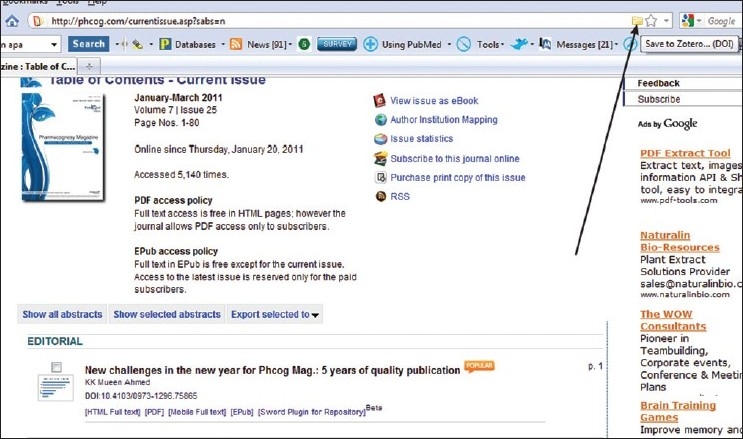
Synchronizing with Zotero – A firefox plugin

**Figure 3:**
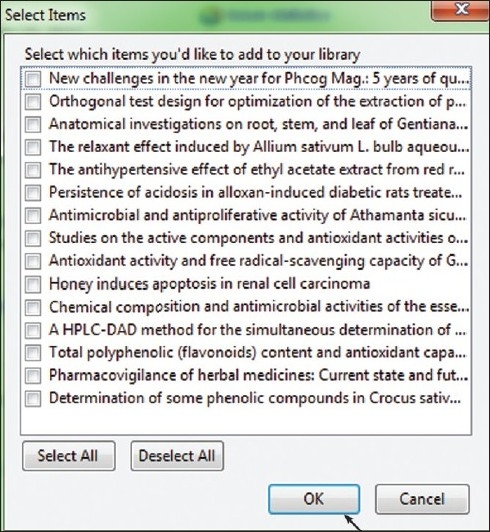
Importing citations

**Figure 4:**
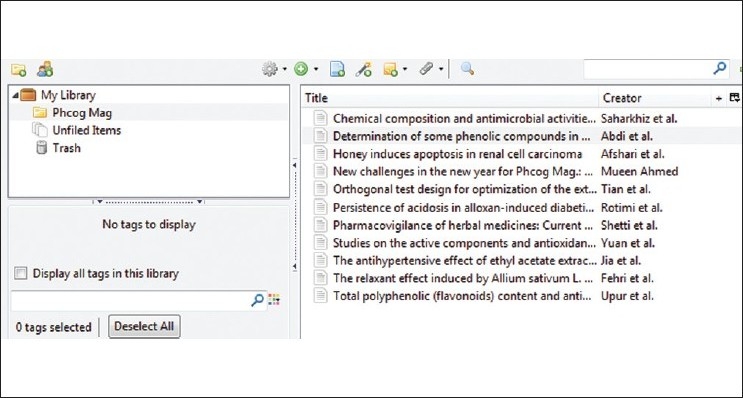
Organizing citations and exporting in different formats using Zotero panel

Zotero is a free citation management tool that works within the Firefox Web browser. Similar to RefWorks and EndNote, Zotero allows you to automatically import citations into your personal account, organize sources into folders, and generate bibliographies in a variety of citation styles. It also includes a word processor plugin for formatting footnotes and parenthetical citations. It easily supports synchronizing articles directly from the *Phcog Mag*. website. Zotero utilizes DOI in retrieving citations and one can easily organize the required citations in different formats and export them based on their need. Figures 2–4 show the various steps involved in automatic synchronizing of *Phcog Mag*. articles with just clicking an icon present in the address bar. Adding *Phcog Mag*. articles is made easy with Zotero (visit www.zotero.org[7] for more details).

## CITING *PHCOG MAG*. WHILE ON GOOGLE SCHOLAR

**Figure 5:**
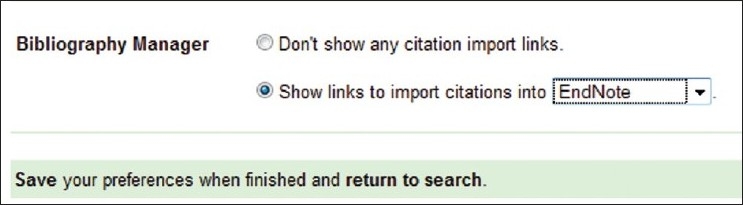
Choosing bibliography manager in Scholar preferences

**Figure 6:**
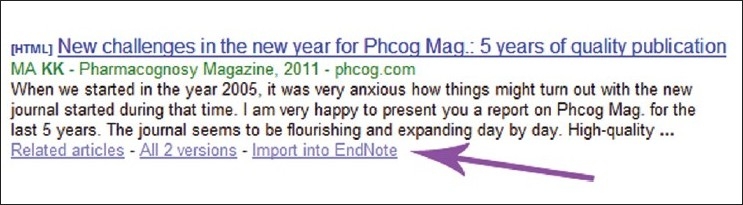
Importing citations in different formats using Google Scholar

Google Scholar is a freely accessible web search engine that indexes the full text of scholarly literature across an array of publishing formats and disciplines. Through its “cited by” feature, Google Scholar provides access to abstracts of articles that have cited the article being viewed. It also shows “Related articles” feature, wherein it presents a list of closely related articles.[8] *Phcog Mag*. article citations appearing in Google Scholar can be downloaded as endnote, refworks, Bibtex file, etc. It can easily be done via changing the required citation format in Scholar preferences [Figures 5 and 6].

To summarize, it is very important that authors understand the relevance of citing quality articles and our commitment to publishing the highest quality of articles along with easily citable formats. I request all the authors and readers to cite and utilize these important medicinal plant researches being published for advancing their research.

Finally, I would like to acknowledge with gratitude the support of Medknow Publications and Media Pvt. Ltd., Mumbai, India, for their excellent productivity.

Thanks, Happy reading!!!
